# Leveraging CD16 fusion receptors to remodel the immune response for enhancing anti-tumor immunotherapy in iPSC-derived NK cells

**DOI:** 10.1186/s13045-023-01455-z

**Published:** 2023-06-14

**Authors:** Fanyi Meng, Siqi Zhang, Juan Xie, Yuan Zhou, Qingling Wu, Binyan Lu, Shixin Zhou, Xiangyu Zhao, Yang Li

**Affiliations:** 1 Department of Cell Biology, School of Basic Medical Sciences, Peking University Stem Cell Research Center, Peking University, Beijing, China; 2 Peking University Institute of Hematology, Peking University People’s Hospital, Beijing, China; 3 Department of Biomedical Informatics, MOE Key Lab of Cardiovascular Sciences, School of Basic Medical Sciences, Peking University, Beijing, China; 4 Guangzhou Regenverse Therapeutics Co.,Ltd., Guangzhou, China

**Keywords:** Induced pluripotent stem cells, NK-cell differentiation, CD16 fusion receptor, Immunotherapy

## Abstract

**Background:**

The cytotoxicity of NK cells is largely dependent on IgG Fc receptor CD16a, which mediates antibody-dependent cell-mediated cytotoxicity (ADCC). The high-affinity and non-cleavable CD16 (hnCD16) is developed and demonstrated a multi-tumor killing potential. However, the hnCD16 receptor activates a single CD16 signal and provides limited tumor suppression. How to exploit the properties of hnCD16 and incorporate NK cell-specific activation domains is a promising development direction to further improve the anti-tumor activity of NK cells.

**Methods:**

To expand the applications of hnCD16-mediated ADCC for NK cell-based immunotherapy in cancer, we designed the hnCD16 Fusion Receptor (FR) constructs with the ectodomain of hnCD16 fused with NK cell-specific activating domains in the cytoplasm. FR constructs were transduced into CD16-negative NK cell line and human iPSC-derived NK (iNK) cells and effective FR constructs were screened. The up-regulation of immune activation- and cytokine-releasing-related pathways in FR-transduced NK cells was screened and validated by RNA sequencing and multiplex cytokines release assay, respectively. The tumor-killing efficiency was tested in vitro and in vivo via co-culture with tumor cell lines and xenograft mice-bearing human B-cell lymphoma, respectively.

**Results:**

We screened the most effective combination to kill B cell lymphoma, which was fused with the ectodomain of hnCD16a, NK-specific co-stimulators (2B4 and DAP10) and CD3ζ in cytoplasmic domains. The screened construct showed excellent cytotoxicity effects and sharp multiple cytokines releasing both in the NK cell line and iNK cells. The transcriptomic analysis and validation assays of hnCD16- and hnCD16FR-transduced NK cells showed that hnCD16FR transduction remodeled immune-related transcriptome in NK cells, where significant upregulation of genes related to cytotoxicity, high cytokines releasing, induced tumor cell apoptosis, and ADCC in comparison with hnCD16 transduction were highlighted. In vivo xenograft studies demonstrated that a single low-dose regimen of engineered hnCD16FR iPSC-derived NK cells co-administered with anti-CD20 mAb treatment mediated potent activity and significantly improved survival.

**Conclusion:**

We developed a novel hnCD16FR construct that exhibits more potent cytotoxicity than reported hnCD16, which is a promising approach to treat malignancies with improved ADCC properties. We also offer a rationale for NK activation domains that remodel immune response to enhance CD16 signaling in NK cells.

**Supplementary Information:**

The online version contains supplementary material available at 10.1186/s13045-023-01455-z.

## Introduction

Adoptive cell therapy using engineered immune effector cells has emerged as a promising new approach to treating hematological and solid malignancies [1, 2]. Among several options of immune effectors, natural killer (NK) cells are proven to play a critical role in the innate immune response to malignant cells and viral infections [3]. Especially, chimeric antigen receptor (CAR)-engineered NK cells have gained considerable attention recently due to their unique biological properties [4, 5]. Unlike T cells, NK cells lack surface T cell receptors (TCRs) and do not cause graft-versus-host disease (GvHD) [6, 7], which is a key advantage of allogeneic NK cell therapies that enables scaled manufacturing and off-the-shelf administration of engineered NK cells for cancer immunotherapy. Clinical trials assessing adoptive transfer of allogeneic NK cells demonstrate that this therapy is safe, with little evidence of toxicities such as cytokine release syndrome (CRS) or neurotoxicity [8, 9]. Moreover, autologous CAR-T cell therapy can persist for many years after transplantation [10], whereas allogeneic NK cells usually survive for a shorter period of time in the host [6, 8, 11]. This property of allogeneic NK cells may facilitate a more accurate dosing strategy, allowing multiple combination dosing, and avoiding long-term cumulative toxicity, which is commonly observed among patients undergoing CAR-T therapy [12].

There are various sources from which NK cells for cancer immunotherapy can be derived, namely peripheral blood-derived NK (PB-NK) cells, umbilical cord blood-isolated NK (UCB-NK) cells, and the NK cell lines. PB-NK and UCB-NK adoptive cell therapies have notched numerous successes in the treatment of various hematologic cancers. However, NK cell yields and subpopulation composition of PB-NK cells and UCB-NK cells are extremely donor-dependent because these cells are often not derived from a single renewable source, making product standardization and multiple-dosing strategies difficult [13, 14]. Additionally, genetic modification of primary NK cells is poorly efficient but highly variable, which makes it difficult to exploit consistent and reproducible engineered NK cell therapies [15]. The immortalized cell line NK-92 would be a competitive alternative as it shows anti-tumor effects comparable to the other NK cell sources while having the adding value of indefinite proliferation [16]. The major disadvantage of NK-92 cells is that, because of their tumorigenic nature and cytogenetic abnormalities, they must be irradiated to suppress their proliferation in the patient prior to clinical use [17]. To this end, induced pluripotent stem cells (iPSCs) have recently emerged as an appealing source for NK cells, given their clonal growth, high expansion capacity, and ability to differentiate in vitro and be genetically modified [18, 19]. These advantages of iPSCs circumvent many of the challenges in NK cell therapy, paving the path for producing a homogeneous engineered NK cell population that can be expanded to clinical scale as an “off-the-shelf” supply.

NK cell-mediated anti-tumor activity is regulated through a repertoire of activating and inhibitory cell surface receptors. Activating receptors of NK cells include CD16, along with natural cytotoxicity receptor family members (NKp30, NKp44, NKp46), and co-stimulatory receptors such as LFA-1, 2B4, and 4-1BB [20, 21]. These activating cell surface receptors have the capacity to trigger cytolytic programs as well as cytokine and chemokine secretion through intra-cytoplasmic immunoreceptor tyrosine-based activation motifs (ITAMs) such as in 2B4 and 41BB and/or through other transmembrane signaling adaptors such as DAP10, CD3ζ, and FcϵRIγ [22–25]. Upon activation, NK cells release cytotoxic granules containing perforin and granzymes to directly lyse tumor cells. CD16a is the most potent activating receptor expressed by NK cells. Of note, CD16a is the only receptor that can activate NK cells on its own, without any additional activation through other receptors [22, 26]. Accordingly, the Fc regions of IgG antibodies on opsonized cells serve to crosslink CD16a molecules and thus activate NK cells through a process termed antibody-dependent cell-mediated cytotoxicity (ADCC). The binding affinity of CD16a to IgG varies between its allelic variants. Specifically, CD16a with valine instead of phenylalanine at position 158 has higher Fc binding affinity and is associated with increased ADCC [27, 28]. Besides, CD16a expression is negatively regulated by the metalloproteinase ADAM17, which cleaves this receptor from the surface of NK cells after they are activated, resulting in decreased CD16a expression [29]. With genetic modification, the ADAM17 cleavage site on CD16a can be mutated to block CD16a shedding and increase ADCC [29]. In iNK cells, a CD16a molecule with the high-affinity F158V mutation and resistance to ADAM17 cleavage (termed hnCD16) could maintain CD16a surface expression and demonstrate increased cytotoxicity in combination with monoclonal antibodies [30, 31].

Here, we hypothesize that hnCD16 fused with NK cell activation domains (hnCD16 fusion receptor, hnCD16FR) would enhance CD16 signaling to improve anti-tumor activity in iNK cells. We screened several constructs and identified one combination (hnCD16-2B4-DAP10-CD3ζ, FR1) mediated a more effective ADCC-induced killing of tumors than hnCD16 in NK cells. Transcriptomic analysis and validation thereof showed that FR1 NK cells showed significant upregulation of genes related to cytotoxicity, high cytokines releasing, induced tumor cell apoptosis, and ADCC compared to hnCD16 NK cells. And more importantly, a single low-dose regimen of engineered FR1 iNK cells administered with anti-CD20 mAb treatment mediated potent activity and improved survival in a mouse xenograft lymphoma model.

## Methods

### Cell lines

Raji and A549 were obtained from the National Infrastructure of Cell Line Resource (Beijing, China) and were engineered to stably express luciferase and green fluorescent protein (GFP). YT cells were kindly provided by Dr. Xiangyu Zhao (Peking University People's Hospital.). YT and Raji cells were cultured in RPMI 1640 medium (Gibco) supplemented with 10% fetal bovine serum (FBS) and 1% penicillin–streptomycin. A549 cells were maintained in DMEM (Gibco) with 10% FBS and 1% penicillin–streptomycin.

### PBMCs isolation and culture

Peripheral blood was collected from one female healthy donor whose written consent was obtained in accordance with the guidelines of the Peking University Health Science Center Ethical Committee. Briefly, 5 mL of peripheral blood was diluted with an equal amount of phosphate-buffered saline (PBS), layered over 10 mL of Ficoll-Hypaque Premium gradient (GE Healthcare), according to the manufacturer's instructions, and centrifuged at 1000 × g for 30 min. The interface that contained mononuclear cells was collected, diluted with a threefold volume of PBS, and centrifuged at 290 × g for 10 min. Peripheral blood mononuclear cells (PBMCs) were grown on six-well plates and maintained in SFM supplemented with the following cytokines: 50 ng/ml SCF, 10 ng/ml IL-3, 2 U/ml erythropoietin (EPO), 40 ng/ml insulin-like growth factor 1 (IGF-1) (all from R&D Systems) for 5–8 days.

### Generation of iPSCs with sendai viral vectors

5 × 10^4^ PBMCs were placed in 1 well of a 12-well plate and infected with the CytoTune-iPS reprogramming kit (Thermo Fisher) containing Sendai virus vectors encoding OCT4, SOX2, KLF4, cMYC in SFM at a multiplicity of infection (MOI) of 5 or 10 for each factor. One day after infection, the cells were harvested and plated onto two wells coated with Matrigel in a six-well plate and cultured in the same medium for an additional day. On day 2, the medium was changed to TeSR-E8 medium (STEMCELL Technologies). Colonies with morphology similar to that of embryonic stem cell-like colonies started to appear on day 13 after infection; they were picked on day 21 or 28 and expanded.

### Molecular constructs and lentivirus production

The lentiviral transfer plasmid was constructed using a typical backbone containing a chimeric 5’ long terminal repeat (LTR), the packaging signal, rev-responsive element (RRE), Woodchuck hepatitis virus posttranscriptional regulatory element (WPRE), and a 3’ self-inactivating (SIN) LTR. hnCD16/FR1/FR2/FR3 was placed under the control of a constitutive promoter (hEF1A). The extracellular domain of CD16a with F158V and S197P site mutation, the transmembrane domain of CD16; cytoplasmic signaling domain of 2B4, DAP10, and CD3Z or FCER1G were used to construct NK-specific fusion receptors. The hnCD16, FR1, FR2 and FR3 constructs are composed of signal peptide of CD16A (FCGR3A) (1-16AA), ectodomain (17-208AA, UniProtKB P08637) with 2 mutations (F158V and S197P) and transmembrane (209-229AA, UniProtKB P08637). The intracellular domains of FR1 are directly fused the cytoplasmic domain of 2B4 (251-370AA, UniProtKB Q9BZW8), DAP10 (70-93AA, UniProtKB Q9UBK5) and CD3ζ (52-164AA, UniProtKB P20963). The intracellular domains of FR2 are fused the cytoplasmic domain of DAP10 (70-93AA, UniProtKB Q9UBK5) and CD3ζ (52-164AA, UniProtKB P20963). The intracellular domains of FR3 are fused the cytoplasmic domain of 2B4 (251-370AA, UniProtKB Q9BZW8) and FCER1G (45-86AA, UniProtKB P30273). The hnCD16 cytoplasmic domain is the nature CD16A (230-254AA, UniProtKB P08637). All of the DNA sequences were synthesized by Syngentech Co., LTD. (Beijing, China). Correct sequences were confirmed by restriction enzyme digest and sequencing analyses. The virus was produced in 293 T cells (ATCC) by co-transfection of the transfer plasmid, a packaging plasmid, and an envelope plasmid. The virus-containing supernatant was harvested on day 2 and day 3 and concentrated before usage.

### Generation of FR-modified NK cells and iPSCs

Control/hnCD16/FR1/FR2/FR3 NK cells were generated by transducing with lentiviral particles expressing Control(eGFP)/hnCD16/FR1/FR2/FR3 at a MOI of 60 with 8 ug/mL polybrene. Transduction efficiency was assessed by flow cytometry on day 4 after transduction. eGFP-positive Control/hnCD16/FR1/FR2/FR3 NK cells were then sorted using the BD FACSAria™ III Cell Sorter for expansion. For generation of Control/hnCD16FR1-iPSCs, iPSCs were transduced with lentiviral particles expressing Control(eGFP)/FR1 construct at a MOI of 20 with 8 ug/mL polybrene. eGFP-positive Control/hnCD16FR-iPSCs were sorted by using the BD FACSAria™ III Cell Sorter and plated at low density for clonal expansion. Selected NK cells and hiPSCs were scaled up, banked, and used for downstream experiments.

### NK cell derivation and expansion from iPSCs

hiPSCs were differentiated first into hematopoietic stem/progenitor cells and then into NK cells as previously described protocol [32]. Briefly, the hiPSCs were cultured using APEL supplemented with 40 ng/ml human stem cell factor (SCF), 20 ng/ml human vascular endothelial growth factor (VEGF), 20 ng/mL recombinant human bone morphogenetic protein 4 (BMP-4) and 10 μM ROCKi (Y-27632). After six days of hematopoietic differentiation, spin embryoid bodies (EBs) were transferred directly into each well of an uncoated 6-well plate under the conditions of NK cell differentiation medium (DMEM/F12 (Gibco), 20% EliteGro-Adv (Elitecell), 1% penicillin–streptomycin-solution (ThermoFisher), 1% GlutaMAX (ThermoFisher), 1 μM β-mercaptoethanol (ThermoFisher), 5 ng/mL sodium selenite (Sigma-Aldrich), 50 μM ethanolamine (MP Biomedicals), 20 mg/mL ascorbic acid (Sigma-Aldrich), 5 ng/mL IL-3 (PeproTech) (first week only), 20 ng/mL SCF (PeproTech), 20 ng/mL IL-7 (PeproTech), 10 ng/mL IL-15 (PeproTech), and 10 ng/mL Flt3 ligand (PeproTech)) Half-media changes were performed weekly for 28–35 days until the cells had developed into CD45^+^CD56^+^CD3^−^ cells as determined by flow cytometry. After assessing NK-cell markers, the cells were expanded and maintained in a medium of RPMI 1640 supplemented with 10% FBS and 1% penicillin–streptomycin-glutamine (ThermoFisher), 50 U/mL IL-2 (Peprotech), and 100 ng/mL IL-21 (Peprotech) with media changes every 3–4 days.

### Xenogeneic lymphoma animal models

To assess the anti-tumor effect of hnCD16FR-transduced iNK cells in vivo, NOD. Cg-Prkdc^scid^ Il2rg^tm1Vst^/Vst (NPG) xenograft model was used, with the aggressive NK-resistant Raji cell line. Mice were given 1 × 10^5^ Luc-expressing tumor cells. For the IV models, Control/hnCD16FR-iNK cells (10^6^ cells per mouse) were injected IV 1 day after tumor cell infusion. Human iPSC-NK cells were supported by the injection of interleukin-2 (IL-2) and/or IL-15. Tumor burden was determined by BLI using a Xenogen IVIS Imaging system. All NPG mice were obtained from Beijing Vitalstar Biotechnology. Mouse experiments were performed in accordance with NIH recommendations under protocols approved by the Institutional Animal Care and Use Committee of Peking University.

### Cytotoxicity assay

To determine the cytotoxicity of YT and iNK cells in vitro, we constructed a lentiviral expression vector expressing luciferase and then transfected the target cells as described above. Control/hncd16/FR1/FR2/FR3 YT or control/hnCD16FR iNK cells and Luc-expressing Raji or A549 tumor cells were incubated in 96-well plates with or without the addition of a therapeutic mAb (Obinutuzumab/Nimotuzumab) and at a defined effector-to-target ratio (E: T) for 12 h. Cytotoxicity was assessed by a standard luciferase-based bioluminescence assay as previously described [33], and the percentage of killing was determined by the following formula: % specific killing = 100 × (spontaneous death RLU − test RLU)/(spontaneous death RLU). RLU: relative luminescence unit.

### Flow cytometry

Cells were stained with antibodies at 1:100 dilution in PBS with 2% FBS for 30 min at room temperature in the dark. Beckman Coulter CytoFLEX and BD FACSAria II were used for flow cytometry analysis and cell sorting, and data were analyzed with FlowJo software and Prism (GraphPad Software). The antibodies are described in Additional file 6: Table S1.

### Multiplex cytokines and secreted proteins analysis

Cytokines and pro-apoptotic factors released by YT and iNK cells were assessed using the LEGENDplex Human CD8/NK Cell Panel (BioLegend) according to the manufacturer's instructions. Briefly, fluorescent beads were incubated with supernatants of 1 × 10^5^ YT or iNK cells co-cultured with Raji tumor cells at a ratio of 1:1, and flow cytometry was used to perform IL-10, IL-6, TNF-α, IFN-γ, granzyme A, granzyme B, perforin, and granulysin in conditioned media for quantitative analyzed with the LEGENDplex™ Data Analysis Software (BioLegend).

### Immunofluorescence staining

Cells were fixed with 4% paraformaldehyde for 10 min at room temperature. After washing with PBS, the cells were treated with PBS containing 10% normal bovine serum albumin (Sigma) and 0.1% Triton X-100 for 30 min at room temperature and then incubated with primary antibodies at 4 °C overnight. Primary antibodies included SSEA-4 (1:100, Santa Cruz Biotechnology), NANOG (1:100, Abcam), OCT4 (1:100, Santa Cruz), SOX2 (1:100, Abcam). Normal mouse or rabbit serum was used as a negative control. Localization of antigens was visualized with anti-rabbit or anti-mouse IgG secondary antibodies conjugated with fluorescein (Santa Cruz).

### Teratomas formation

Animal experiments were performed in accordance with the guidelines of the Institutional Animal Care and Use Committee of Peking University. Male NOD-SCID mice (4–6 weeks of age) were purchased from the Department of Experimental Animal, Peking University Health Science Center. iPSCs were harvested by EDTA solution treatment, suspended in PBS, and subcutaneously injected into the thighs of mice. After about 6 weeks, teratomas were explanted, fixed in 4% paraformaldehyde, embedded in paraffin, and examined histologically using immunohistochemistry staining.

### RNA-sequencing and data analysis

Total RNA samples isolated from CTRL/hnCD16/FR1/FR2 YT cells were treated with DNase using a column assay. High-quality RNA (280/260 and 230/260 both over 1.7, RIN/RQN > 9) was subjected to ribosomal RNA depletion followed by library construction. For routine gene expression analysis, raw reads were quality-controlled and adaptor-pruned by SOAPnuke (v1.5.6). The clean reads were mapped to the human genome (GRCh38.p13), by HISAT2 (v2.1.0). Gene expressions were quantified by RSEM (v1.3.1) tool. Differentially expressed genes (DEGs) were identified by DESeq2(v1.4.5) and visualized by Pheatmap (1.0.8) software packages in R. The threshold for DEG was |log2FC|≥ 1 and *Q*-value (corrected *P* value) ≤ 0.05. Functional enrichment analysis of DEGs was performed by Phyper (https://en.wikipedia.org/wiki/Hypergeometric_distributionopen in new window) based on the Hypergeometric test, and GSEA software was used to analyze genes function from the MSigDB database. *Q*-values were used to assess the significance.

### Statistical analysis

Data are shown as mean ± standard deviation of the mean (SD). In vitro data were obtained from 3 independent experiments. Differences between groups were assessed using one-way ANOVA and two-way ANOVA. For quantification of in vivo images, data are expressed as mean ± SEM, and differences between groups were analyzed using a two-tailed Student's *t*-test. Survival curves were analyzed using the log-rank (Mantel–Cox) test. All statistical analysis was performed using GraphPad Prism. It was considered significant at *p* < 0.05 for all tests.

## Results

### Screening-efficient construct that improves cytotoxicity to tumors by fusing NK activating domains and hnCD16 ectodomain

**Fig. 1:**
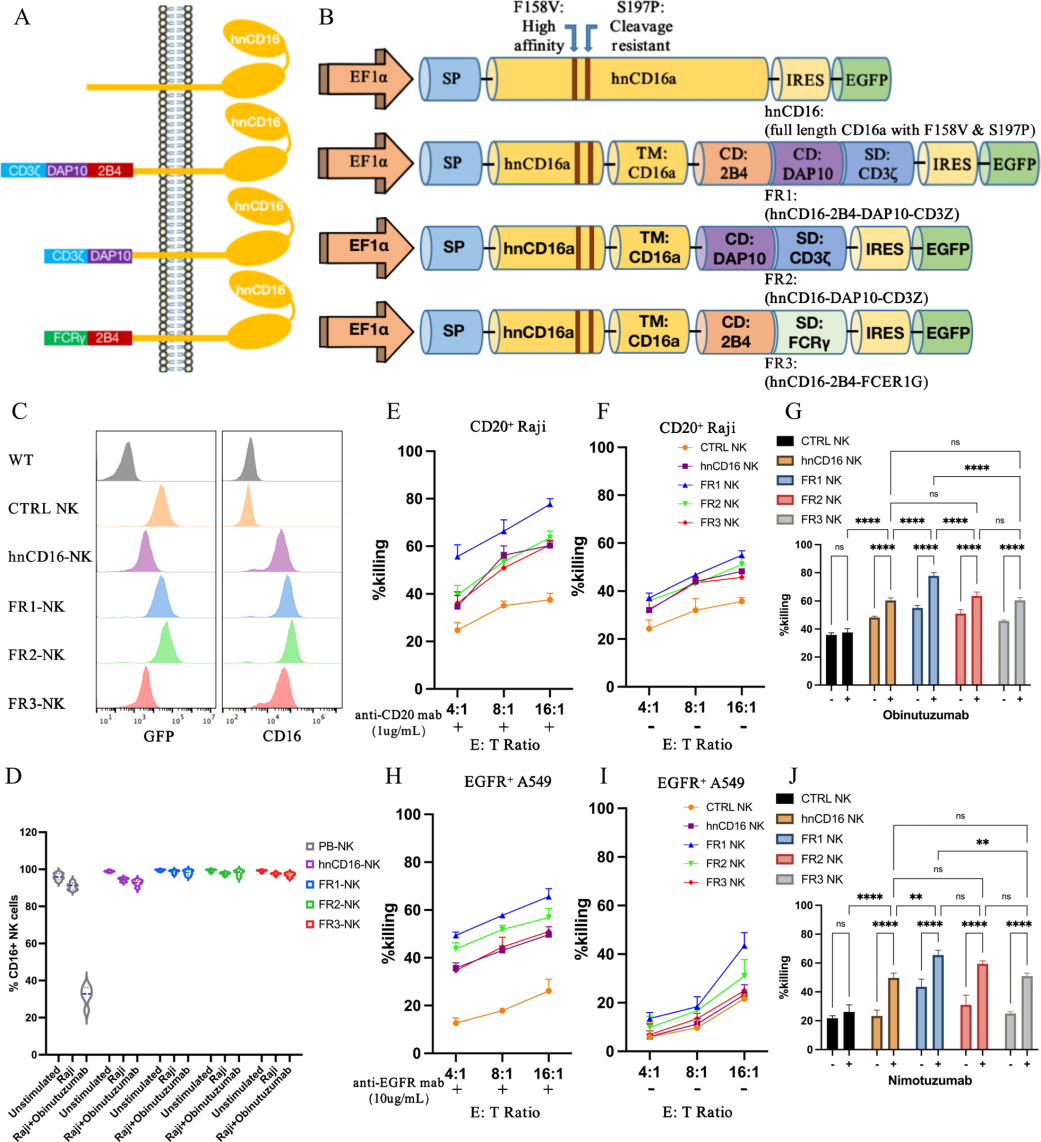
CD16-based fusion receptor constructs mediated anti-tumor activity in NK cells. **A**–**B** Schematic representation of the vector structure encoding the fusion receptor. Transmembrane (TM), co-stimulating domain (CD), stimulation domain (SD). **C** Expression of GFP and surface expression of CD16 in NK cells. **D** PB-NK cells, hnCD16-NK cells, or hnCD16FR-NK cells were stimulated for 6 h, and expression of the CD16 was determined by flow cytometry (*n* = 5 per group). **E**–**G** NK cells that express the designed FRs were co-cultured with Raji cells, with **E** or without **F** anti-CD20 antibody, for 12 h. ADCC was analyzed using a standard luciferase-based bioluminescence assay. The mean of percentages of specific killing ± SD are shown. **G** Statistical significance at 16:1 E:T ratio was determined by two-way ANOVA. Results are representative of three independent experiments. *p* > 0.05 (ns), *p* ≤ 0.05 (*), *p* ≤ 0.01 (**), *p* ≤ 0.001 (***), *p* ≤ 0.0001 (****). **H**–**J** NK cells that express the designed FRs were co-cultured with A549 cells, with (**H**) or without (**I**) anti-EGFR antibody, for 12 h. ADCC was analyzed using a standard luciferase-based bioluminescence assay. The mean of percentages of specific killing ± SD is shown. **J** Statistical significance at 16:1 E:T ratio was determined by two-way ANOVA. Results are representative of three independent experiments. *p* > 0.05 (ns), *p* ≤ 0.05 (*), *p* ≤ 0.01 (**), *p* ≤ 0.001 (***), *p* ≤ 0.0001 (****)

Three different fusion receptor constructs were screened in our initial study and tested for their activating effect on NK cell function. All fusion receptors were designed based on CD16a variants that can increase the affinity of FcγRIIIa for the Fc fragment of the antibody (F158V) and resist ADAM17-mediated cleavage (S197P) [27, 34], and were optimized for enhancing the activity in NK cells. We designed NK cell-specific hnCD16 fusion receptor constructs that consist of the signal peptide, the extracellular and transmembrane domain of hnCD16, intracellular 1 or 2 co-stimulatory domains (CDs) typically expressed in NK cells as well as stimulatory domain (SD) CD3ζ or FcϵRIγ (Fig. 1A, B). CD16a does not have any ITAM structural domain in its cytoplasmic tail and therefore requires the help of intracellular chain tandems containing ITAM structural domains CD3ζ and FcϵRIγ [35, 36], which are both associated with CD16a [37].

Initially, we chose to express these fusion protein constructs in NK cell line YT (which is not expressing CD16a) to test whether these NK cells engineered with hnCD16FR could increase the ability to kill tumor cells. These constructs could be stably expressed on NK cell membrane at comparable levels (Fig. 1C). To assess the stability of hnCD16FR expression, we activated hnCD16-NK, hnCD16FR-NK and PB-NK cells using different combinations of stimuli (Fig. 1D). The results showed that after stimulation by tumor cells and Obinutuzumab, PB-NK cells lost most of their CD16 expression, whereas NK cells expressing hnCD16 and hnCD16FR could remain CD16 expression at high levels (Fig. 1D). Next, the engineered NK cells were tested for their ability to kill Raji cells expressing CD20 receptor under conditions with or without the anti-CD20 mAb at different effector-to-target (E: T) ratios (Fig. 1E–G). Administration of anti-CD20 mAb induced a significant increase in ADCC-mediated antitumor capacity in NK cells expressing hnCD16FR and hnCD16 at low, medium, and high ratios (Fig. 1E). Remarkably, NK cells expressing FR1 (hnCD16-2B4-DAP10-CD3ζ) construct had the greatest increase in their natural cytotoxicity and ADCC in the absence or presence of anti-CD20 mAb. Interestingly, NK cells expressing hnCD16FR and hnCD16 showed an enhancement in their antitumor activity upon stimulation with tumor cells in the absence of anti-CD20 mAb, compared to the control cells (Fig. 1F).

To verify that hnCD16FR combined with different therapeutic mAb can cause effective killing of cancer cells expressing different tumor-associated antigen, we performed ADCC assay on lung cancer cell line A549 using NK cells expressing each of the three hnCD16FR constructs. Similar to the results for killing Raji cells, the FR1 (hnCD16-2B4-DAP10-CD3ζ) construct showed superior ADCC effects on A549 cells over the other constructs (Fig. 1H–J). FR2 (hnCD16-DAP10-CD3ζ) and FR3 (hnCD16-2B4-FcϵRIγ) constructs mediated comparable killing of A549 cells with the hnCD16 construct.

Our data demonstrate that hnCD16 fused with the combination of 2B4-DAP10-CD3ζ facilitated the activation of ADCC function to enhance antibody-induced NK cell-mediated antigen-dependent antitumor cytotoxicity, compared to the hnCD16 construct previously reported [30].

### Expression of hnCD16 fusion protein in NK cells remodels immune-related transcriptome in NK cells

**Fig. 2:**
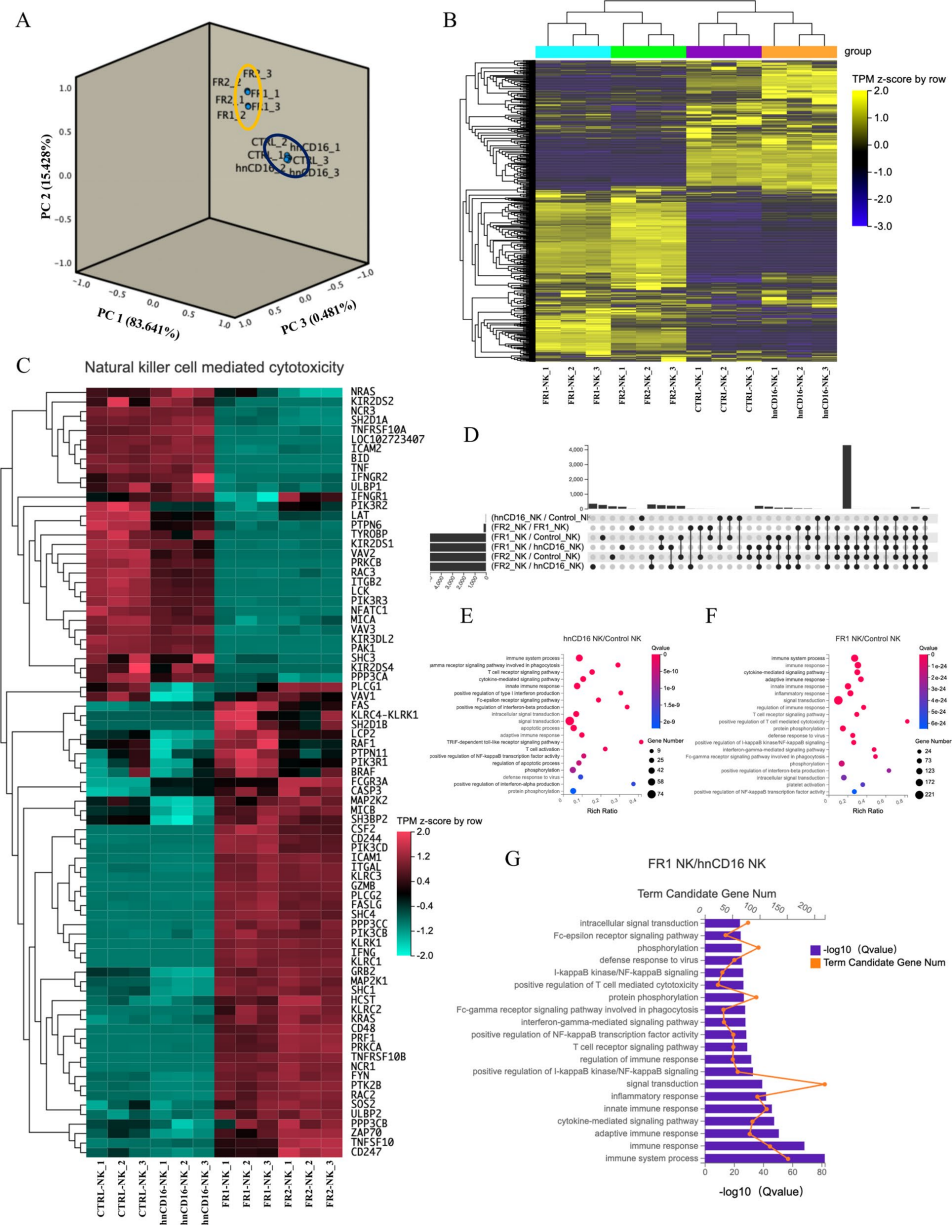
The hnCD16 fusion receptors induce alterations in the transcriptome of NK cells. **A** Principal component analysis of gene expression analyses of the indicated NK cell populations. **B** Heatmap showing unsupervised clustering analysis based on immune function-related genes (*n* = 3). **C** Unsupervised hierarchical clustering analysis based on genes associated with NK cell-mediated cytotoxic pathways. **D** Upset plot showing the numbers and overlaps between differentially expressed genes (DEGs) in various inter-group comparison sets. Each row indicates a DEG set, while each column indicates a combination of DEG sets, as indicated by the linked dots. The bar plot on the left side represents the number of genes of each DEG set and that on the upper side represents the size of overlap between different DEG sets. **E**–**G** Top GO terms of biological processes enriched in DEGs between hnCD16 NK cells versus Control NK cells (**E**), FR1 NK cells versus Control NK cells (**F**), and FR1 NK cells versus hnCD16 NK cells (**G**)

We next performed transcriptomic sequencing (RNA-seq) to compare overall gene expression differences between CTRL (eGFP), hnCD16, FR1 (hnCD16-2B4-DAP10-CD3ζ), and FR2 (hnCD16-DAP10-CD3ζ) transduced NK cells in the absence of stimuli. In the principal component analysis (PCA) result of the transcriptomic data, the FR1 and FR2 NK cell populations formed one cluster, separating from another cluster formed by CTRL- and hnCD16-transduced cells (Fig. 2A). Inter-sample expression profile correlation analysis and unsupervised hierarchical clustering of expression profiles of immune-related genes showed a similar pattern (Additional file 1: Fig. S1A; Fig. 2B), where FR1 NK cells and FR2 NK cells had similar immune gene expression profiles that were distinct to those of control NK cells and hnCD16 transduced cells.

We further compared the expression of genes more specifically associated with NK cell-mediated cytotoxic pathways (Fig. 2C). Interestingly, NK cells transduced with hnCD16FR showed significant upregulation of genes associated with NK cell immune response such as CD48, ICAM1, GZMB, IFNG, TNFSF10, and NKG2 family proteins, in addition to demonstrating activation of CD16 downstream signaling genes CD247, ZAP70, and PI3K. Meanwhile, expressions of TNF and TNFRSF10A, which encode TNF-α and TRAILR1, respectively, were down-regulated in FR1 and FR2 groups. The IFNγ receptors IFNGR2, as well as MICA, a ligand of NKG2D, were also down-regulated in FR1 and FR2 groups. These results indicate that NK cells transduced with FR exhibited an increased gene expression profile for the activation of immune function and NK cell-mediated cytotoxic pathway (Fig. 2B, C).

We further performed Gene Ontology (GO) functional enrichment analysis of inter-group DEGs (Fig. 2D–H). We observed that the Fc-γ receptor signaling pathway was enriched in DEGs between hnCD16-expressing NK cells and control NK cells (Fig. 2E). In addition, significant pathway enrichment occurred in the immune response, cytokine-mediated signaling pathway, and innate immune response in DEGs between FR1/FR2-expressing NK cells and control NK cells (Fig. 2F; Additional file 1: Fig. S1B). Notably, adaptive immune response, inflammatory response, and T cell receptor signaling pathways were also enriched in DEGs between FR1/FR2 NK cells and hnCD16-expressing NK cells (Fig. 2G; Additional file 1: Fig. S1C). We observed that genes in the pathways related to adaptive immune response and cytokine secretion were activated in FR1/FR2 NK cells, while many genes encoding functions that determine NK cell activation and effector functions were highly induced in both FR1/FR2 NK cells and adaptive NK cells (Fig. 2C, G; Additional file 1: Fig. S1C). We reasoned that these cells may have undergone transcriptional changes toward adaptive or memory-like NK cells which have a superior adaptive capacity to environmental changes and immune functions.

**Fig. 3:**
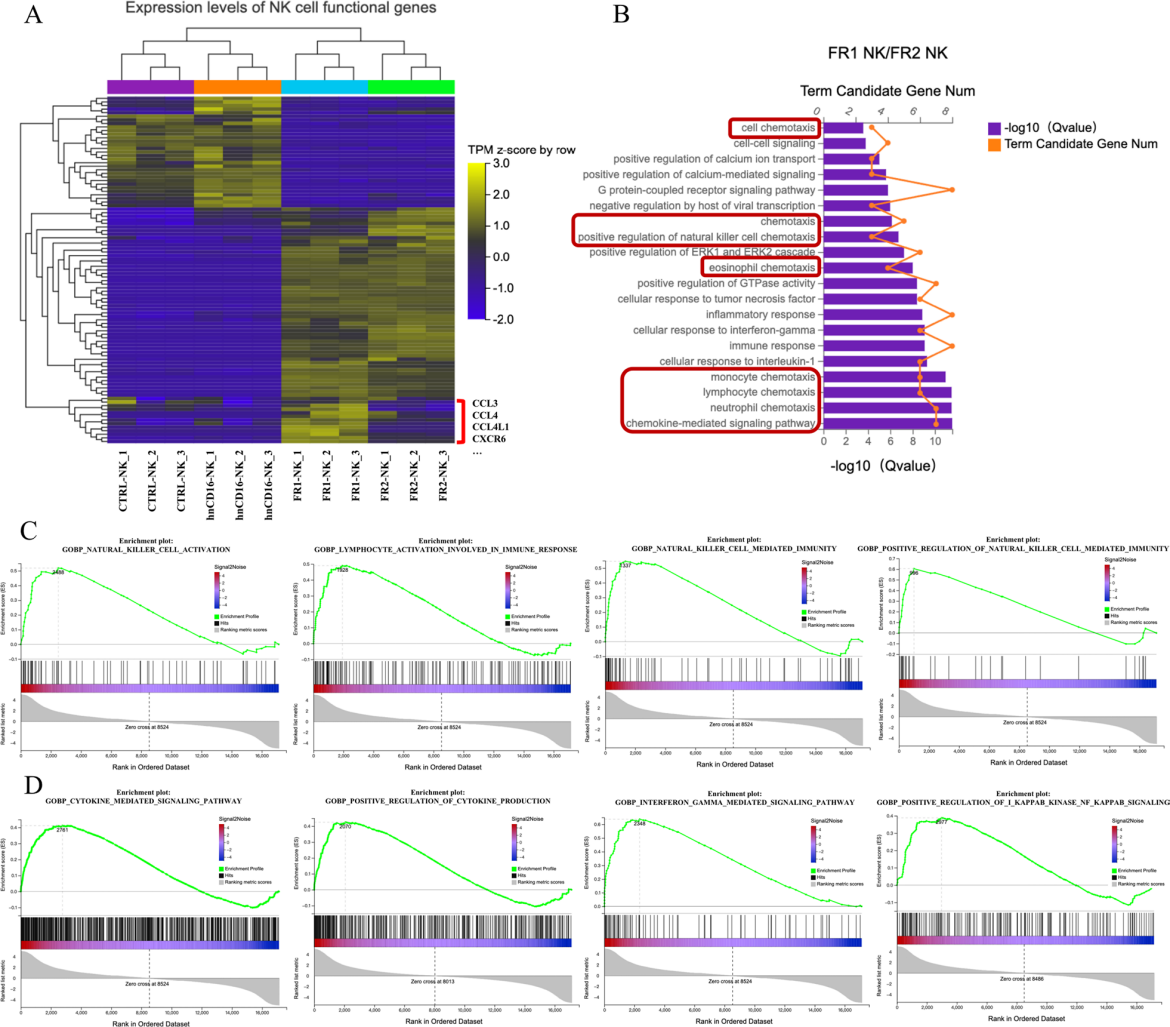
FR1 construct induces a broad upregulation of gene expression related to NK function. **A** Unsupervised clustering analysis showing expression levels of NK cell functional genes across all clusters. Red square bracket highlights up-regulated genes in FR1 NK cells in comparison with FR2 cells. **B** Top GO terms of biological processes enriched in FR1 NK cells versus FR2 NK cells by NK functional upregulation gene analysis (*n* = 3). Red frames highlight the biological processes related to chemotaxis. **C** and **D** GSEA analysis identifying up-regulated pathways related to NK cell function (**C**) and cytokine secretion (**D**) in FR1 NK cells in comparison with hnCD16 NK cells

Furthermore, to distinguish FR1 from FR2 construct, we performed transcriptomic analysis of immune-related functional genes in NK cells expressing FR1 (hnCD16-2B4-DAP10-CD3ζ) and FR2 (hnCD16-DAP10-CD3ζ), and a set of NK cell functional genes were significantly up-regulated in FR1 by unsupervised clustering analysis (Fig. 3A), and a further GO analysis of these upregulated genes revealed that these genes were enriched in chemokine-mediated signaling pathway (Fig. 3B), with notable upregulation of gene transcripts for CCL3, CCL4, and CXCR6 (Fig. 3A). The result suggests that NK cells expressing FR1 not only possess the capability to produce and secrete high levels of antitumor cytokines but also develop the potential to transform into memory-like NK cells in the adaptive immune response (Fig. 3B). Finally, because GO enrichment analysis did not directly imply the up-/down-regulation of the associated pathways, we also performed Gene Set Enrichment Analysis (GSEA) by comparing the expression profiles in FR1 NK cells in comparison with hnCD16 NK cells and observed that genes associated with NK cell activation and cytokine secretion tend to be significantly up-regulated in the FR1 group (Fig. 3C, D).

### hnCD16FR-expressing NK cells exhibit more robust and enhanced cytokine release in vitro

**Fig. 4:**
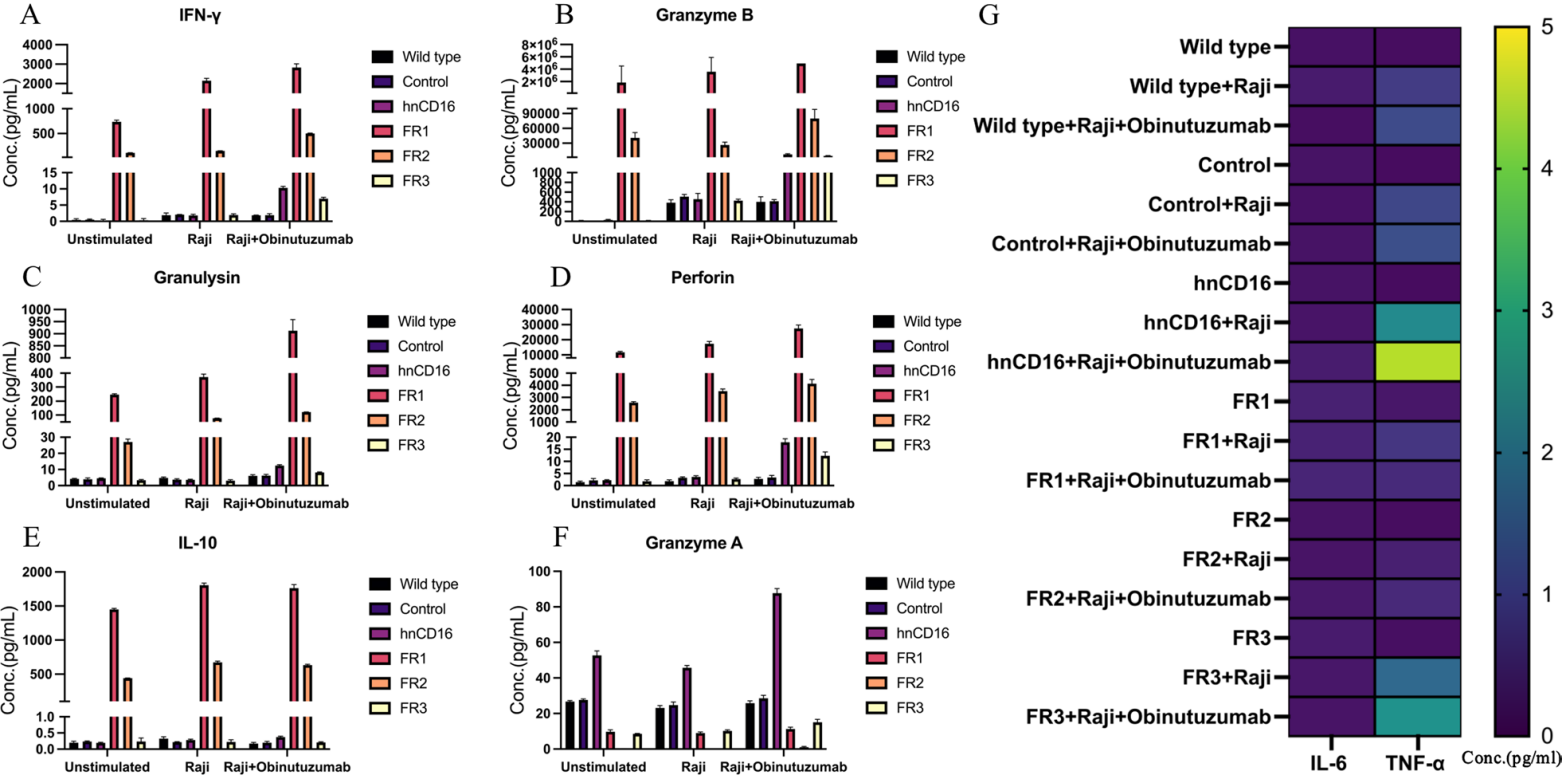
Cytokine secretion profile of hnCD16FR-NK cells. **A**–**F** Quantification of effector cytokine production by flow cytometry analysis after incubation of NK cells with medium (unstimulated) or with indicated stimuli. **G** Heatmap quantifies IL-6 and TNF-a production and scales from 0 pg/ml (purple) to 5 pg/ml (yellow). Studies were repeated three times independently. The mean of concentration (Conc.) ± SD is shown

To verify the up-regulation of genes related to cytokine production in hnCD16FR-expressing NK cells, we further measured the secretion capacity of pro-inflammatory and pro-apoptotic cytokines using beads-based multiple cytokine release assays. We observed that upon stimulation with tumor cells or tumor cells plus Obinutuzumab, the secretion of IFN-γ, Granzyme B, Perforin, Granulysin, and IL-10 was significantly increased in NK cells expressing hnCD16FR (Fig. 4A–E), whereas Granzyme A was secreted at high levels only in NK cells expressing hnCD16 (Fig. 4F). Of note, FR1- and FR2-expressing NK cells maintained high levels of cytokine secretion even in the absence of stimulation, which we consider to be beneficial for sustaining their efficient tumor-killing function. In contrast, a low level of TNF-α was detected in hnCD16FR-expressing NK cells (Fig. 4G), which may be associated with the immunomodulation of IL-10 that can intensely inhibit the synthesis and release of TNF-α inflammatory factors at the transcriptional level [38]. IL-6 secretion was almost undetectable in all NK groups (Fig. 4G). In summary, NK cells expressing hnCD16FR1/FR2 could mediate enhanced anti-tumor cytokine release than NK cells expressing hnCD16, while no statistically significant increase was observed in NK cells expressing FR3. This data indicates that an appropriate hnCD16FR construct is important to improve and enhance antibody-dependent cytokine responses against NK-resistant tumor cells.

### Generation of iPSCs stably expressing hnCD16FR

iPSC-derived NK (iNK) cells can be produced in a homogenous manner and are capable of being genetically modified at the iPSC stage. Therefore, our goal and strategy are to differentiate iPSCs engineered with our lead construct hnCD16FR1 to iNK cells for off-the-shelf NK cell therapy to treat malignancies. For the generation of iPSCs, PBMCs were transduced using Sendai viruses encoding human OCT4, SOX2, KLF4, and c-MYC genes. On approximately 18 days after transduction, the colonies containing 10–20 cells with a high nucleus-to-cytoplasm ratio could be observed under bright-field microscopy. Over the next weeks, the size of colonies increased gradually (Additional file 2: Fig. S2). On about day 28 post-transduction, individual colonies were lifted from the plate and then were passaged by 0.5 mM EDTA solution for expansion. Immunohistochemistry showed that iPSC colonies were positive for OCT4, SOX2, NANOG, and SSEA-4 (Additional file 3: Fig. S3A), which are pluripotency markers extensively used for assessing the characteristics of iPSCs [39]. To test pluripotency in vivo, we transplanted human iPSCs subcutaneously into SCID mice. Six weeks after injection, palpable bulges under the skin were observed. Histological examination showed that the tumor contained derivatives from three germ layers including neural epithelium, muscle, and glandular epithelium (Additional file 3: Fig. S3B).

**Fig. 5:**
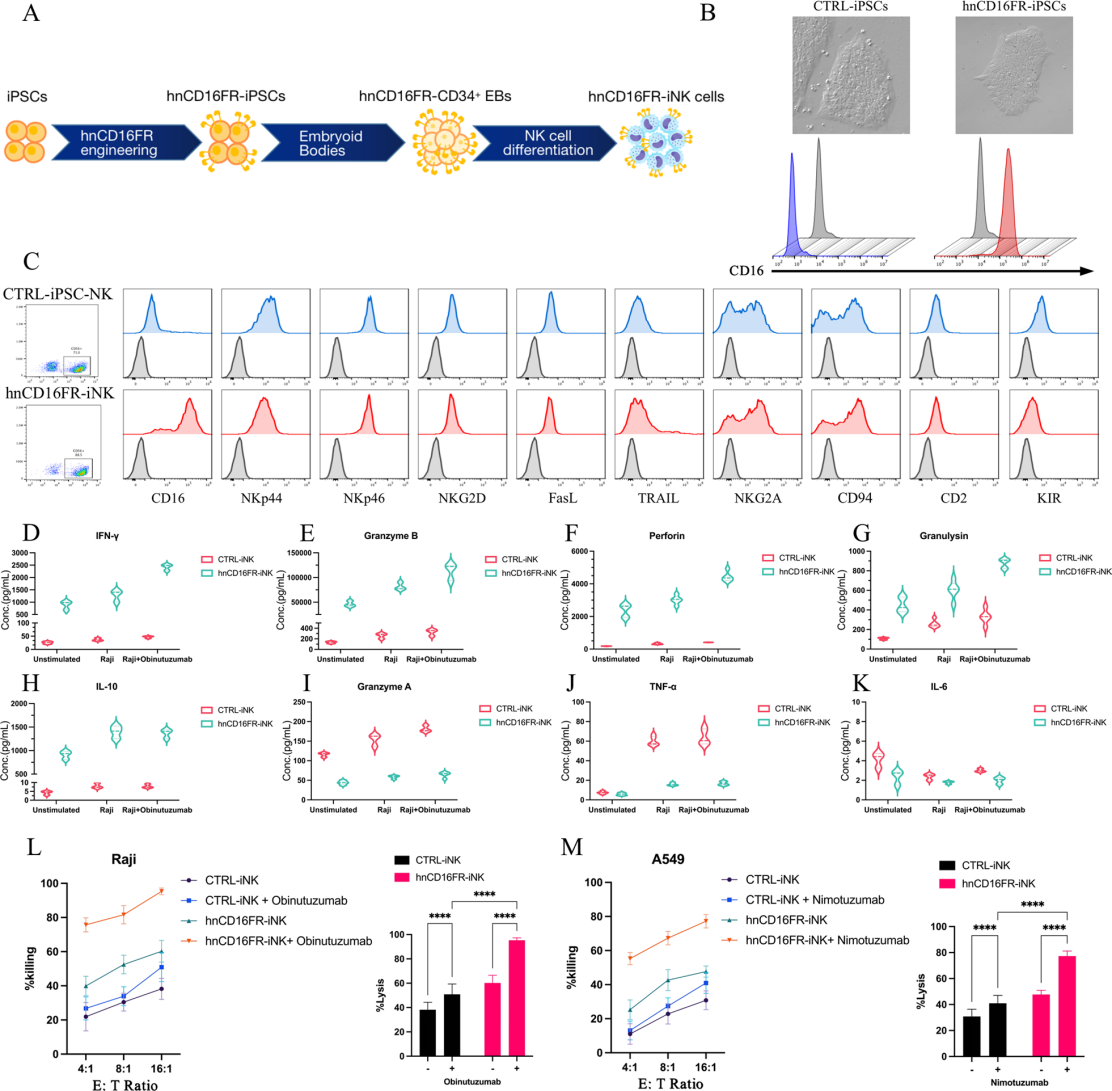
Phenotypic characterization of iPSC-derived NK cells expressing hnCD16FR and anti-tumor function via ADCC. **A** Schematic illustration of the steps leading from human iPSCs to hnCD16FR-iNK cells. **B** Representative images showing CTRL(eGFP)-iPSCs and hnCD16FR-expressing iPSCs (up) and CD16 expression (down) by flow cytometry. **C** Flow cytometry analysis of NK cell surface receptors in the gate of CD56^+^ NK cell populations. In each panel, dark line: isotype control; blue/red line: stained sample. Data were repeated independently in three separate experiments. **D**–**K** Violin plots of cytokine secretion levels in CTRL(eGFP)-iNK cells and hnCD16FR-iNK cells. Cytokines release was assessed using the flow cytometry analysis. Data were repeated independently in three separate experiments. **L**–**M** ADCC assays using a standard luciferase-based bioluminescence assay. ADCC against the lymphoma cell line Raji with/without anti-CD20 mAb (**L**) and against the lung cancer cell line A549 with/without anti-EGFR mAb (**M**). Statistical significance at 16:1 E:T ratio was determined by two-way ANOVA. Results are representative of three independent experiments; *p* > 0.05 (ns), *p* ≤ 0.05 (*), *p* ≤ 0.01 (**), *p* ≤ 0.001 (***), *p* ≤ 0.0001 (****)

To maintain stable hnCD16FR expression, we transduced human iPSCs with Control (eGFP) and hnCD16FR lentivirus and sorted positive cells for clonal expansion. From the transduced hnCD16FR pool, clonal iPSC lines were screened to ensure homogeneous origin material (Fig. 5A). Flow cytometry analysis showed that the clonal iPSC cell lines engineered with hnCD16FR could stably express hnCD16FR, and iPSCs engineered with hnCD16FR maintained typical undifferentiated morphology (Fig. 5B).

### hnCD16FR-iPSC-derived phenotypically mature NK cells exhibit enhanced and superior ADCC-mediated antitumor activity

We then conducted NK differentiation in vitro using a previously reported protocol [32] (Additional file 4: Fig. S4). Phenotypically, both Control iNK cells and hnCD16FR-expressing iNK cells expressed similar levels of typical NK cell surface antigens such as NKp44, NKp46, NKG2D, NKG2A, TRAIL, and FasL, together with NK cell maturation markers (e.g., CD94 and CD2) (Fig. 5C). Notably, the expression of CD16 was observed in almost all hnCD16-iNK cells, whereas endogenous expression of CD16 on Control iNK cells was on average at a lower level under the same culture condition (Fig. 5C). This phenomenon is consistent with previously reported studies where endogenous CD16 on the surface of NK cells was cleaved by ADAM17 upon activation by stimuli (e.g., cytokines or target cells) [29].

We first examined the cytokine release of iNK cells under various conditions using multiple cytokine release assays (Fig. 5D–K). As expected, Control iNK cells secreted IFN-γ, granzyme B, Perforin, Granulysin, and IL-10 at low levels without stimulation. These cells did not exhibit a significant increase in the cytokine release when co-cultured with Raji cells, or Raji cells plus anti-CD20 mAb (Fig. 5D–H), whereas Granzyme A and TNF-α release significantly increase upon stimulation in Control iNK cells (Fig. 5I–J). In contrast, we observed that hnCD16FR-expressing iNK cells maintained the secretion of IFN-γ, Granzyme B, Perforin, Granulysin, and IL-10 at high levels without stimulation (Fig. 5D–H). Upon stimulation, these cytokine productions sharply increased, while Granzyme A and TNF-α release kept at low levels in hnCD16FR-expressing iNK cells (Fig. 5I–J). Both Control and hnCD16FR-expressing cells have very low IL-6 secretion with or without stimulation. Interestingly, the phenotype of multiple cytokine release in iNK cells was consistent with that observed in the NK cell line. Overall, the above data suggest that the introduction of hnCD16FR structure can markedly enhance the antibody-dependent cytokine secretion response against tumor cells.

To evaluate the effect of antibody-dependent activation-based hnCD16FR-mediated ADCC on different cancer cell types, we investigated the killing ability of engineered hnCD16FR-iNK cells against tumor cell lines including Raji (NK-resistant Burkitt's lymphoma) cells and A549 (lung adenocarcinoma) cells. Antibodies that recognize antigens on these tumor cell lines (Raji cell surface antigen CD20; A549 cell surface antigen EGFR) by combination are clinically approved for use and proven to be effective [40–42]. Similar to the results for cytokine release, Control-iNK cells have limited killing ability against tumor cells, even with the addition of the corresponding therapeutic monoclonal antibody. In contrast, in the presence of anti-CD20 mAb or anti-EGFR mAb, hnCD16FR-iNK cells co-cultured with specific antigen-expressing tumor cells induced a significant increase in proportion to the tumor cells killed (Fig. 5L–M), exhibiting a remarkable enhancement of antigen-specific cell lysis activity. Together, these results demonstrate that hnCD16FR-expressing iPSC-derived NK cells mediate sharp cytokine release as well as improved killing activity against tumor cells, which at least partly can be ascribed to the improved ADCC properties combined with mAb.

### A single low dose of hnCD16FR-expressing iNK cells exhibits more effective ADCC-mediated durable antitumor responses in an in vivo Raji Lymphoma model

**Fig. 6:**
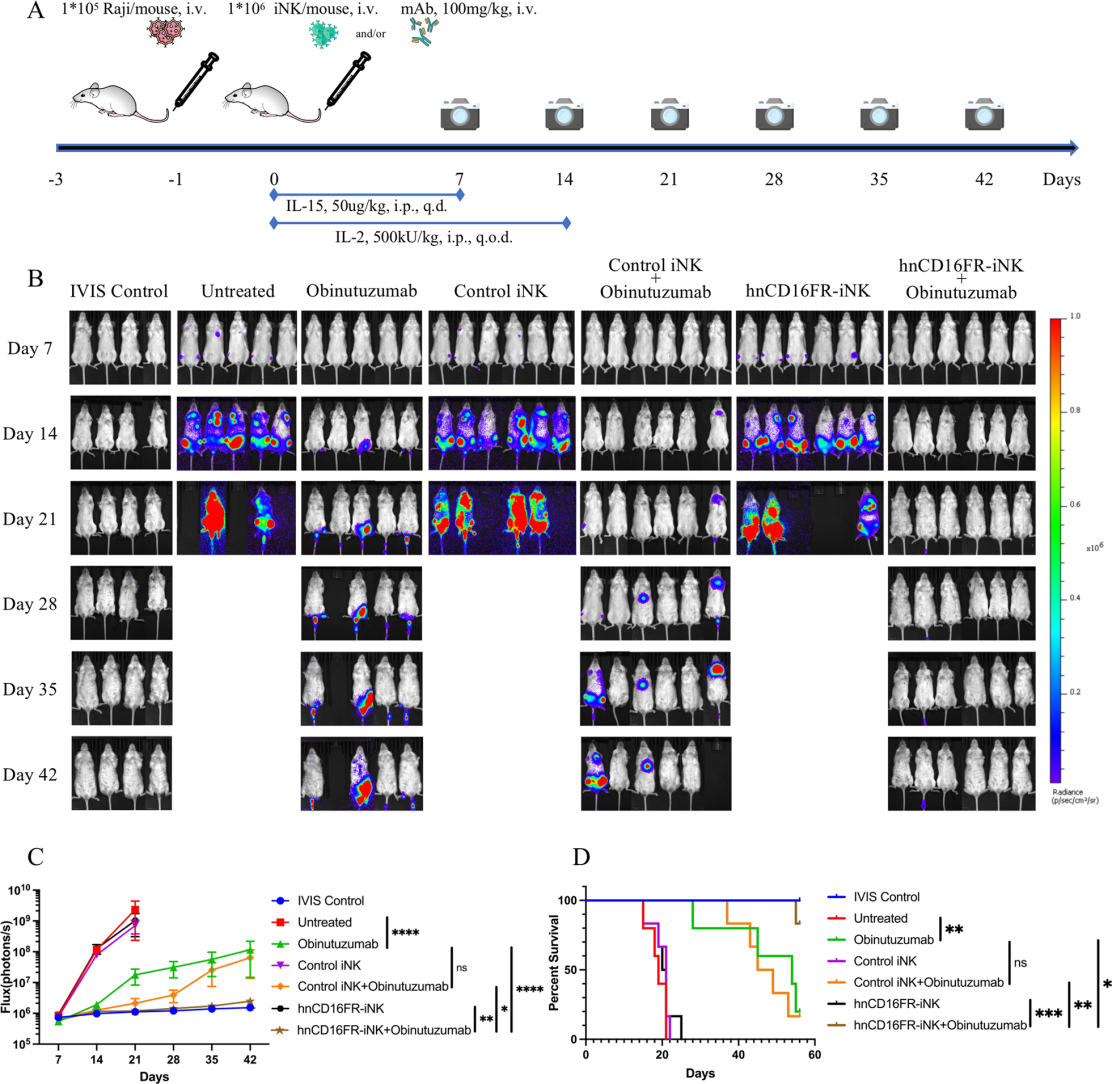
hnCD16FR-iNK cells effectively mediate ADCC for anti-tumor responses in an in vivo Raji lymphoma model. **A** Schematic of the experimental design to test in vivo anti-tumor function of hnCD16FR-iNK cells using luciferase-expressing Raji cells in a mouse xenograft model treated with Obinutuzumab and iPSC-NK populations and cytokine administration. NPG mice were inoculated IV with 1 × 10^5^ Luc-expressing Raji cells. On day 1 after transplant, mice were left untreated (*n* = 5), received IV injection of Obinutuzumab alone (*n* = 5), or were treated with 1 × 10^6^ Control(eGFP) iNK cells alone (*n* = 6), hnCD16FR-iNK cells alone (*n* = 6), or 1 × 10.^6^ Control(eGFP) iNK cells in combination with 100 mg/kg of Obinutuzumab (*n* = 6), hnCD16FR-iNK cells in combination with 100 mg/kg of Obinutuzumab (*n* = 6). NK cells were supported by injection of IL-15 for the first week and by injection of IL-2 for 2 weeks; IVIS imaging was performed weekly to track tumor progression. i.v., intravenously; i.p., intraperitoneally; q.d., every day; q.o.d., every other day. **B** Tumor burden was determined by weekly bioluminescent imaging (BLI). **C** BLI data of the tumor burden of each group were monitored for 42 days after immune effector cell infusion. The BLI data are plotted, and mean ± SD are shown. Statistical significance was determined by two-tailed Student’s *t*-test; *p* > 0.05 (ns), *p* ≤ 0.05 (*), *p* ≤ 0.01 (**), *p* ≤ 0.001 (***), *p* ≤ 0.0001 (****). **D** Kaplan–Meier curve representing the percent survival of the experimental groups: tumor only or treated with Control(eGFP)-iNK cells, hnCD16FR-iNK cells. Untreated versus Obinutuzumab, *p* = 0.0051; Obinutuzumab versus Control(eGFP) iNK+Obinutuzumab, *p* = 0.7989; Obinutuzumab versus hnCD16FR-iNK+Obinutuzumab, *p* = 0.0228; Control(eGFP) iNK+Obinutuzumab versus hnCD16FR-iNK+Obinutuzumab, *p* = 0.0096; hnCD16FR-iNK versus hnCD16FR-iNK+Obinutuzumab, *p* = 0.0006. Statistical significance was determined using a Log-rank (Mantel–Cox) test. *p* > 0.05 (ns), *p* ≤ 0.05 (*), *p* ≤ 0.01 (**), *p* ≤ 0.001 (***)

To assess the in vivo ADCC-mediated anti-tumor activity of the hnCD16FR-expressing iNK cells combined with therapeutic monoclonal antibodies, we prepared Raji xenograft systemic tumor model in severely immunodeficient NOD. Cg-Prkdc^scid^ Il2rg^tm1Vst^/Vst (NPG) mice. Raji-Luc tumor cells, Obinutuzumab, and iNK cells were injected intravenously (i.v.) (Fig. 6A). It is noteworthy that, in this study, without reducing the number of Raji cells used for modeling [30, 43, 44], the single dose used for each mouse is 1 × 10^6^ iNK cells, which is significantly lower than the dose (1 × 10^7^ to 2 × 10^7^) used in previous studies [25, 30, 31, 44]. 24 h after the tumor cells infusion, each group of mice received a single injection of Obinutuzumab alone, a single intravenous injection of Control/hnCD16FR iNK cells alone, or a single intravenous injection of Obinutuzumab plus Control/hnCD16FR iNK cells. Tumor growth was subsequently monitored by weekly tracking to assess changes in tumor BLI for 6 weeks (Fig. 6B–D).

Tumor bioluminescence increased rapidly in the untreated group of mice, with a median survival of 18 days (Fig. 6D). Consistent with previous studies, injection of therapeutic monoclonal antibodies alone demonstrated single-agent anti-tumor activity in immunodeficient mice [30]. However, mice administered Control or hnCD16FR iNK cells alone exhibited only modest tumor inhibition at the early time points, and were unable to constantly suppress tumor growth in the Raji xenograft model (Fig. 6C, D). Combination treatment with Control iNK cells with Obinutuzumab did not significantly relief tumor burden in comparison with treatment with Obinutuzumab alone (Fig. 6C; *p* = 0.1350), while both groups could extend the median survival from 18 to 44 days compared to the untreated group (Fig. 6D).

Importantly, combination treatment with hnCD16FR-iNK cells and anti-CD20 mAb exhibited more potent anti-tumor effects, controlling tumor expansion (Fig. 6B, C) and prolonging survival (Fig. 6D) more than Control iNK cells co-administered with anti-CD20 mAb. The tumor burden was significantly lower than the Obinutuzumab alone treatment group (Fig. 6C; *p* < 0.0001) and the Control iNK cells combined with Obinutuzumab treatment group (Fig. 6C; *p* = 0.0235).

It is also worth noting that five mice treated by the hnCD16FR-iNK cell co-administration with Obinutuzumab maintained complete tumor remission to the experimental endpoint after tumor transplantation, whereas most mice in the other groups failed to survive to the experimental endpoint (Fig. 6D). These results further confirm that engineered iNK cells with hnCD16FR in co-administration with Obinutuzumab can mediate ADCC potently in vivo and provide more durable suppression and clearance of human lymphomas.

## Discussion

iPSCs can now be routinely generated from a variety of easily obtainable sources such as peripheral blood (PB); once reprogrammed, iPSCs are able to undergo essentially unlimited expansion in vitro without losing pluripotency [39]. Therefore, NK cells generated from iPSCs have emerged as a promising strategy to produce standardized, off-the-shelf NK cells for cancer immunotherapy. Like PB-NK cells, iPSC-derived NK (iNK) cells also exhibit cytotoxicity against diverse cancer cells via releasing perforins and granzymes, producing proinflammatory IFN-γ and TNFα, and inducing apoptosis through TRAIL and Fas-FasL interaction [45]. iNK cells were shown to be equally or more effective than PB-NK cells. Hermanson et al*.* demonstrated iNK cytotoxicity was comparable to PB-NK in the in vivo ovarian cancer xenograft models [46]. Zeng et al*.* showed that iNK cells have greater cytotoxicity against multiple cancer cell lines compared to donor PB-NK cells [47]. Here, we reprogrammed peripheral blood mononuclear cells (PBMCs) into iPSCs using Sendai virus expressing reprogramming factors, then efficiently differentiated them into NK cells in a feeder-free manner that express mature NK-cell markers and exhibited cytotoxicity against human tumor cells. Therefore, we can utilize this efficient NK differentiation platform to produce engineered homogeneous human NK cells from iPSCs for cancer immunotherapy.

CD16a engagement provides a potent stimulus to activate NK cells [48]. The clinical anti-tumor activity of monoclonal antibody therapy is in part dependent on NK cell ADCC activity [49]. The high-affinity CD16 variant (F158V) has been shown to lead to improved anti-tumor responses in patients treated with monoclonal antibodies [50, 51]. Additionally, the substitution of the serine at position 197 for a proline (S197P) effectively blocked CD16a cleavage by the metalloprotease ADAM17 from the surface of activated NK cells [34]. The high-affinity and non-cleavable CD16a (termed hnCD16) are developed via two-point mutations (F158V and S197P) to enhance the affinity to the Fc fragment and resist cleavage to ADAM17 protease. Recently, Dan Kaufman's team reported that in iPSC-derived NK cells, hnCD16 maintained CD16a surface expression and demonstrated increased cytotoxicity and IFN-γ production in combination with monoclonal antibodies [30].

Here, we designed NK cell-specific activating domains in cytoplasm fused with the ectodomain of hnCD16 and tested the function of several constructs in CD16-negative NK cell line. Among these constructs, we identified 2B4-DAP10-CD3ζ linking hnCD16 (hnCD16FR) as the best structure compared to other constructs. Previous studies have shown that NK cell activation domains can stimulate cellular immune signaling pathways that mediates critical NK cell effector activity [22, 24, 52, 53]. The NK-specific co-stimulatory structure-mediated signaling is superior to that of NK cells using T cell activation structural domains [25]. Endogenous 2B4 relies on binding to CD48 to trigger phosphorylation of ITAM, a signal that can simultaneously enhance other NK cell receptor-induced activation [54] and is required for NK cell function [55]. The cytoplasmic domain of DAP10 contains the YINM sequence that, when phosphorylated, is able to bind to the p85 subunit of PI3K or the adaptor Grb2 to initiate cytolytic activity [56], and DAP10 is the only option for human NKG2D association [57, 58]. CD3ζ is an important adaptor protein for the induction of ADCC upon CD16 binding to immunoglobulin G (IgG) [35]. The fusion of the hnCD16 extracellular domain and cytoplasmic NK-specific co-stimulatory domains facilitates the activation of NK cells, enhancing cytotoxicity and ADCC effects against tumor-specific antigens. Specifically, hnCD16FR significantly increased the release of cytokines associated with enhanced NK cell immunity. The features of cytotoxicity, ADCC activity, and cytokine releases were significantly increased when immunotherapeutic antibodies existed in tumor-killing assays. The hnCD16FR modification was also shown similar properties in iPSC-derived NK cells in cytotoxicity and cytokine release. The mechanism of NK cells engineered with hnCD16FR was shown transcriptomic remodeling by analysis of immune-enriched genes that are similar to the activating NK cells via RNA-sequencing. Furthermore, the iPSC-derived hnCD16FR NK cells showed significant enhancement of ADCC-mediated cytotoxicity and co-administered with antibodies against NK cell-resistant lymphomas in immunodeficiency mice. All these experiments displayed the distinguished structure of hnCD16FR that significantly enhanced the efficacy of immunotherapy against refractory lymphomas both in vitro and in vivo. Compared to either hnCD16 or FR2 (hnCD16-DAP10-CD3ζ) and FR3 (hnCD16-2B4-FcϵRIγ), FR1 (hnCD16-2B4-DAP10-CD3ζ) could mediate significantly superior ADCC effects in different tissue-derived tumor cell lines. However, these data present in this study are not enough to support the claim that both 2B4 and DAP10 are required for the enhanced cytotoxicity activity of hnCD16 NK cells. To support this statement, we need to design more FR constructs and make detailed comparisons among them. Our data merely suggest that FR1 is an optimal construct to enhance cytotoxicity activity of hnCD16 NK cells among three FR constructs.

Conventional CARs contain only a scFv as an extracellular antigen recognition domain, which has the limitation of binding only a single target antigen. In some recent studies, universal CAR (UniCAR) T cell therapies using adaptor molecules showed some promising results [59, 60], but the use of such approaches requires additional quality control steps for junction molecules and is accompanied by the risk of increased immunogenicity. It takes great advantage of engineering on NK cells for their innate responses to multiple foreign antigens and shorter survival time compared to CAR-T cells. The CD16- or hnCD16-based fusion receptors can be used to target multiple antigens directly by utilizing clinically approved monoclonal antibodies and ADCC-mediated anti-tumor activity for their effectiveness. Meanwhile, these fusion receptors can be terminated by discontinuing administration, as demonstrated in previous studies that cytotoxicity induced by CD16 depends on binding to target cells with specific antibodies [61].

In previous studies, a unique phenomenon was observed that CAR-T cells exhibit elevated basal activation levels independent of antigen engagement when CARs are anchored on the cell surface, which is termed tonic signaling [62, 63]. The underlying mechanism of this phenomenon remains to be further investigated, but it has been reported that CARs may integrate into the TCR-CD3 complex through their CD3ζ domains and may enhance T cell activation [64]. It has also been demonstrated that the CD28 transmembrane domain in some CAR constructs mediates heterodimerization of CARs with endogenous CD28, leading to T cell activation [65]. In this study, we found that FR1- and FR2-modified NK cells produce cytokines even without stimulation, indicating that this increase of basal activation without CD16a engagement may result from tonic signaling. These findings suggest that FRs are not static on the cell membrane but may interact with endogenous receptors. Although the exact nature of these receptor associations in NK cells is unknown, we speculate that FRs may interact synergistically with activating receptors on the NK cell membrane, resulting in enhanced immune activation. We detected the secretion of several cytokines including IFN-γ, Granzyme B, Perforin, Granulysin, and IL-10 in high levels without stimulation in FR1-iNK cells. On the one hand, high level of cytokine secretion even in the absence of stimulation is beneficial for sustaining their efficient tumor-killing function. On the other hand, this will raise a concern about the safety issue of hnCD16FR-engineered iNK cell infusions such as cytokine release syndrome (CRS). CRS is a common adverse reaction of CAR-T cell therapy, which is mainly mediated by pro-inflammatory cytokines such as IL-1, IL-6 and TNF-α [66]. We observed very low IL-6 and TNF-α secretion with or without stimulation in hnCD16FR-iNK cells. And our results also showed a significant increase of the immunoregulatory cytokine IL-10 secretion in hnCD16FR-iNK cells, which can inhibit the synthesis and release of inflammatory cytokines such as IL-1, IL-6, IL-8, TNF-α, GM-CSF and G-CSF, thus balancing immune activation and anti-inflammation [66]. Our histopathological analysis on various tissues from mice treated by hnCD16FR-iNK cell infusion showed no significant evidence of tissue damage (Additional file 5: Fig. S5), hinting that hnCD16FR-iNK treatment has low toxicity in NPG xenografts animal model. These points may mitigate to some extend safety issue about application of hnCD16FR-iNK cells. However, it still should be noted that a limitation of this study is that the high levels of cytokine secretion by hnCD16FR-iNK cells without antigen exposure may pose safety risks due to excessive immune system activation, and this treatment carries the risk of serious adverse events associated with ADCC, although these risks can be reduced by pausing antibody administration or reducing the number of hnCD16FR-iNK cells infused. A second approach to safety would be to add a control switch to minimize the risk associated with adoptive cell transfer therapy and infuse cells with dose-escalation during cell therapy. Besides, one of the properties of NK cells is that they have a limited lifespan and persist in vivo for a short period of time [21, 67], which is a double-edged sword for the hnCD16FR1-iNK cells. On the one hand, a short survival time may weaken continuous anti-tumor activity in vivo. On the other hand, it is more beneficial for the safety of iNK adoptive transfer therapy.

## Conclusions

In summary, our results demonstrate the feasibility to use human iPSCs as a platform to create homogeneous hnCD16FR-expressing NK cells that can be combined with mAb to trigger potent ADCC and multiple cytokine production in response to cancer cells. These hnCD16FR engineered iNK cells can mediate superior Burkitt’s lymphoma control in vivo, as demonstrated in the xenogeneic adoptive transfer experiments.

## Supplementary Information

**Additional file 1. Fig. S1**: Sample correlation and Gene Ontologyenrichment analysis of FR2-NK differentially expressed genes. The heat map reflects the Pearson correlation coefficient of all gene expressions between each pair of samples. Top GO terms of biological processes enriched in FR2 NK cells versus Control NK cells DEGs, and FR2 NK cells versus hnCD16 NK cells DEGs.

**Additional file 2. Fig. S2**: Cell images during induction of iPSCs from PBMCs.Image of PBMCs. Image of attached cells on day 5 post-viral transduction.Image of attached cells on day 12 post-viral transduction. Image of small iPSC colony on day 18 post-viral transduction.Image of iPSC colony on day 22 post-viral transduction. Image of iPSC colony on day 28 post-transduction. Scale bars: 50 μm.

**Additional file 3. Fig. S3**: Expression of pluripotent makers and teratoma formation of PBMC-iPSCs.Immunofluorescence staining of iPSC colonies was positive for OCT4, NANOG, SOX2, SSEA-4.Hematoxylin–eosin staining of a teratoma derived from iPSCs including neural rosettes, muscle, gut-like epithelial tissue. Scale bars: 50 μm.

**Additional file 4. Fig. S4**: Cell images during differentiation of NK cells from human pluripotent stem cells at different stages.

**Additional file 5. Fig. S5**: Representative images of H&E-stained sections of five major organs 7 days after iNK cell infusion. Representative tissue images of kidney, lung spleen, liver, and heart histopathology from mice receiving only tumor cells, control iNK cells, or hnCD16FR iNK cells. Scare bar is shown as 100 μm.

**Additional file 6. Table S1. **Antibodies used in this study.

## Data Availability

All data are available in the main text or in the supplementary materials. All reagents reported in the paper may be available to the scientific community. RNA-seq data are available in the GEO database (GSE223656).

## Abbreviations

iPSC: Induced pluripotent stem cell
ADCC: Antibody-dependent cell-mediated cytotoxicity
hnCD16: High-affinity and non-cleavable CD16
FR: Fusion receptor
iNK: IPSC-derived NK
CAR: Chimeric antigen receptor
TCR: T cell receptor
GvHD: Graft-versus-host disease
CRS: Cytokine release syndrome
PB: Peripheral blood
UCB: Umbilical cord blood
ITAM: Immunoreceptor tyrosine-based activation motif
PBMC: Peripheral blood mononuclear cell
EB: Embryoid body
NPG: NOD. Cg-Prkdc^scid^ Il2rg^tm1Vst^/Vst
RLU: Relative luminescence unit
E:T: Effector-to-target ratio
CTRL: Control
CD: Co-stimulatory domain
SD: Stimulatory domain
PCA: Principal component analysis
GO: Gene ontology
GSEA: Gene set enrichment analysis
BLI: Bioluminescence imaging
EGFR: Epidermal growth factor receptor
Fas-L: Fas ligand
IFN-γ: Interferon-γ
scFv: Single-chain antibody variable fragment
TNF-α: Tumor necrosis factor-alpha
TRAIL: TNF-related apoptosis-inducing ligand
TAA: Tumor-associated antigen
UniCAR: Universal CAR
