# Supplementary figures and images for: Leveraging CD16 fusion receptors to remodel the immune response for enhancing anti-tumor immunotherapy in iPSC-derived NK cells
Source: J Hematol Oncol. 2023 Jun 14;16:62. doi: 10.1186/s13045-023-01455-z (PMC10265820; doi:10.1186/s13045-023-01455-z)

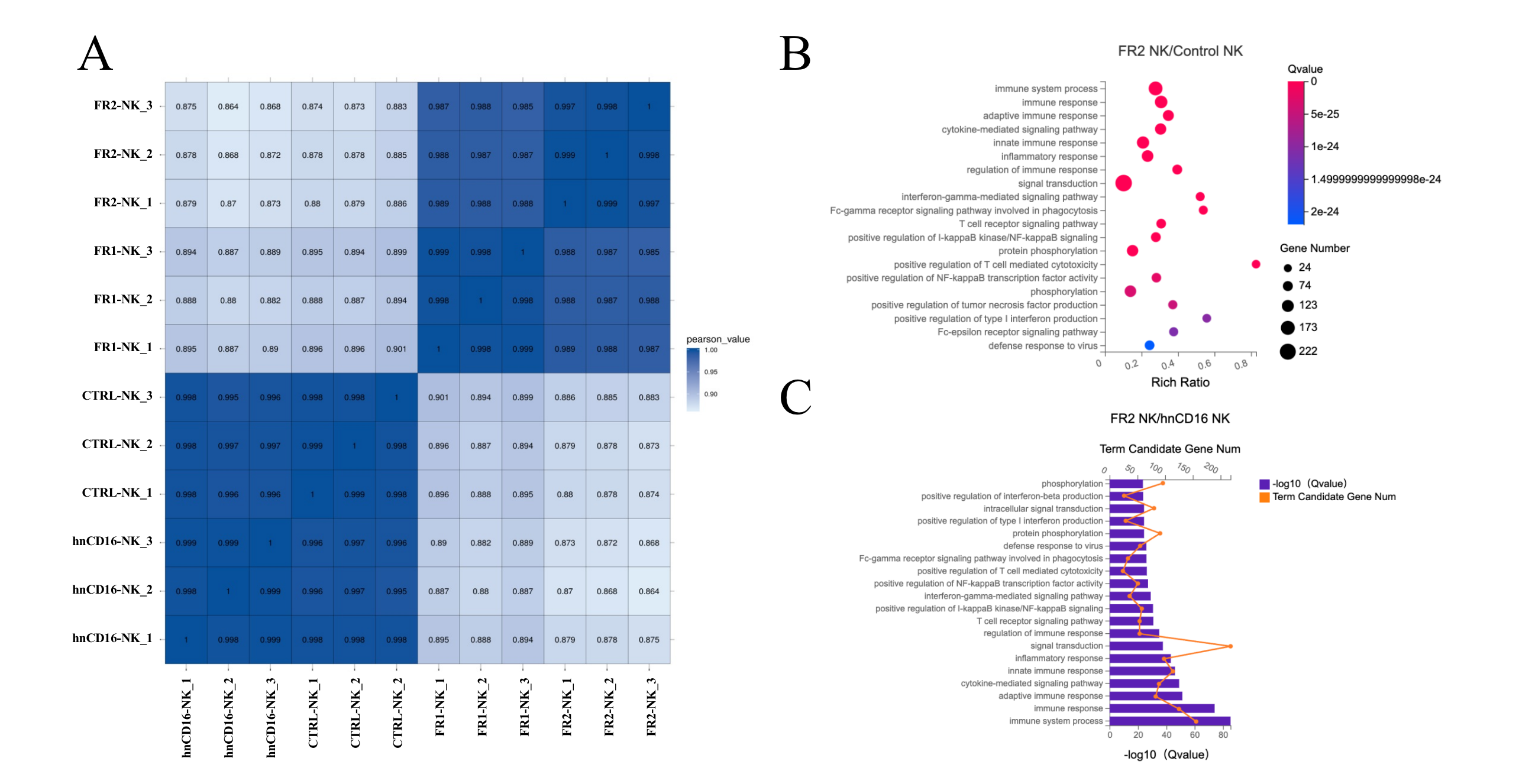

Supplement: Supplementary file 1 — Additional file 1. Fig. S1: Sample correlation and Gene Ontologyenrichment analysis of FR2-NK differentially expressed genes. The heat map reflects the Pearson correlation coefficient of all gene expressions between each pair of samples. Top GO terms of biological processes enriched in FR2 NK cells versus Control NK cells DEGs, and FR2 NK cells versus hnCD16 NK cells DEGs. [file 13045_2023_1455_MOESM1_ESM.png]

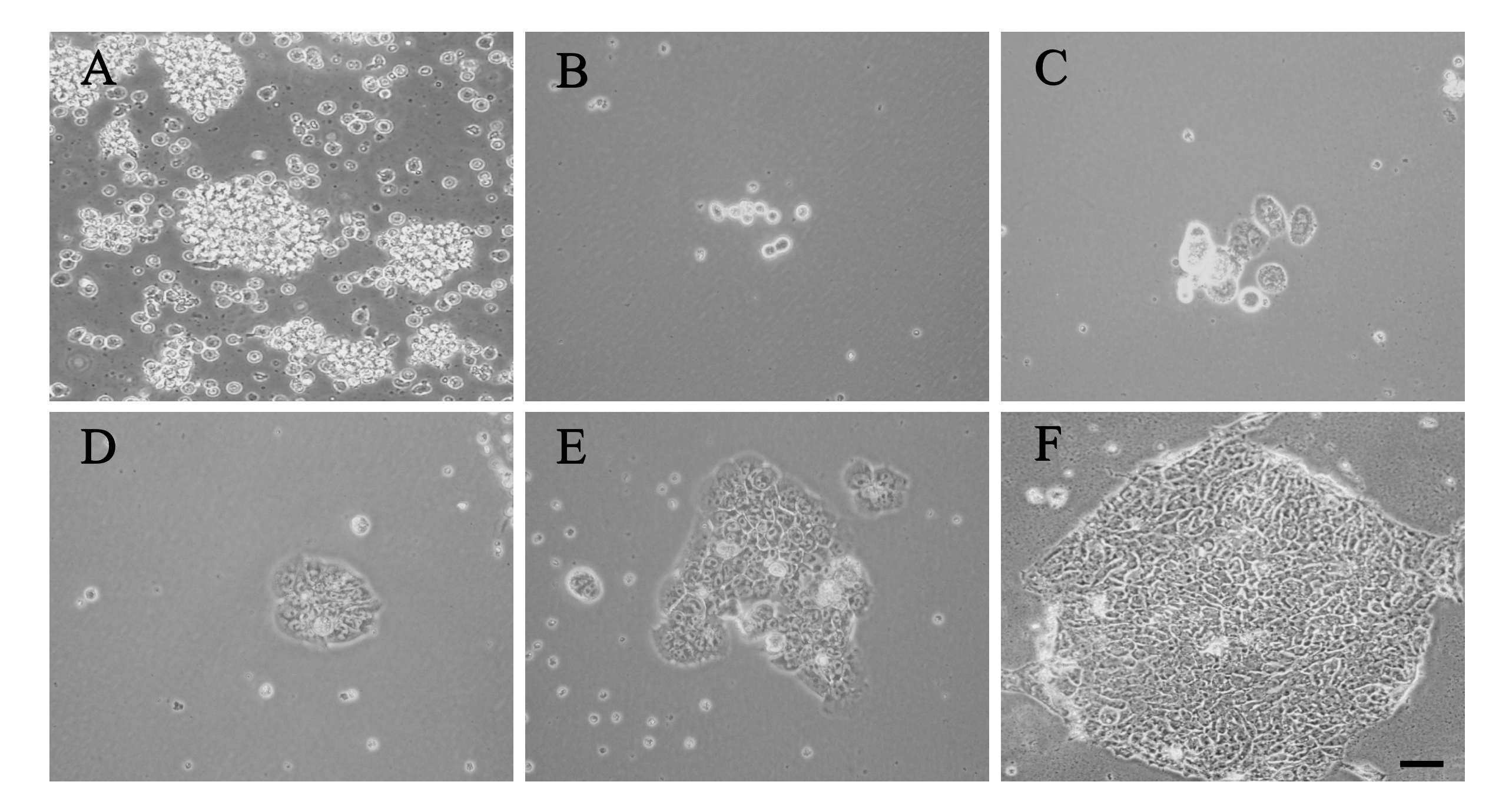

Supplement: Supplementary file 2 — Additional file 2. Fig. S2: Cell images during induction of iPSCs from PBMCs.Image of PBMCs. Image of attached cells on day 5 post-viral transduction.Image of attached cells on day 12 post-viral transduction. Image of small iPSC colony on day 18 post-viral transduction.Image of iPSC colony on day 22 post-viral transduction. Image of iPSC colony on day 28 post-transduction. Scale bars: 50 μm. [file 13045_2023_1455_MOESM2_ESM.png]

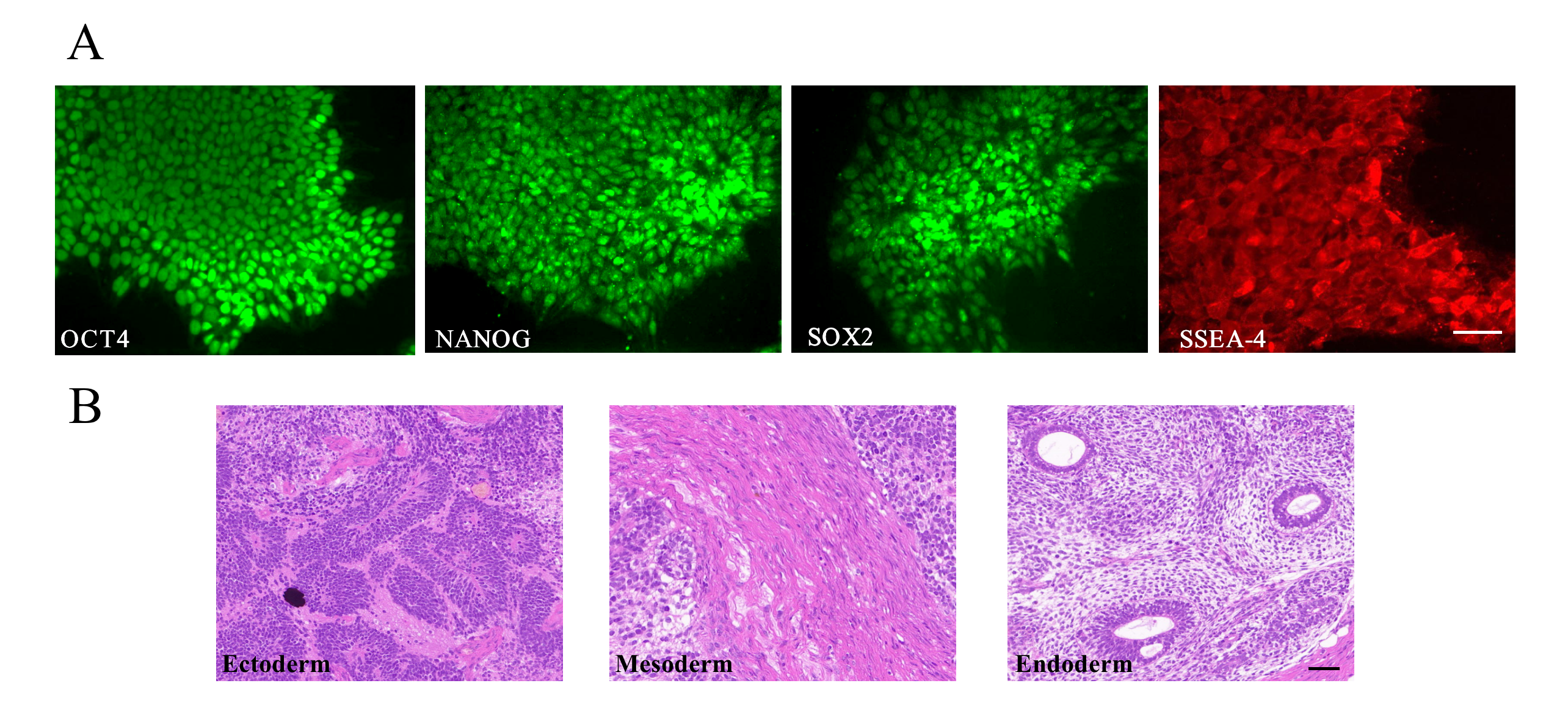

Supplement: Supplementary file 3 — Additional file 3. Fig. S3: Expression of pluripotent makers and teratoma formation of PBMC-iPSCs.Immunofluorescence staining of iPSC colonies was positive for OCT4, NANOG, SOX2, SSEA-4.Hematoxylin–eosin staining of a teratoma derived from iPSCs including neural rosettes, muscle, gut-like epithelial tissue. Scale bars: 50 μm. [file 13045_2023_1455_MOESM3_ESM.png]

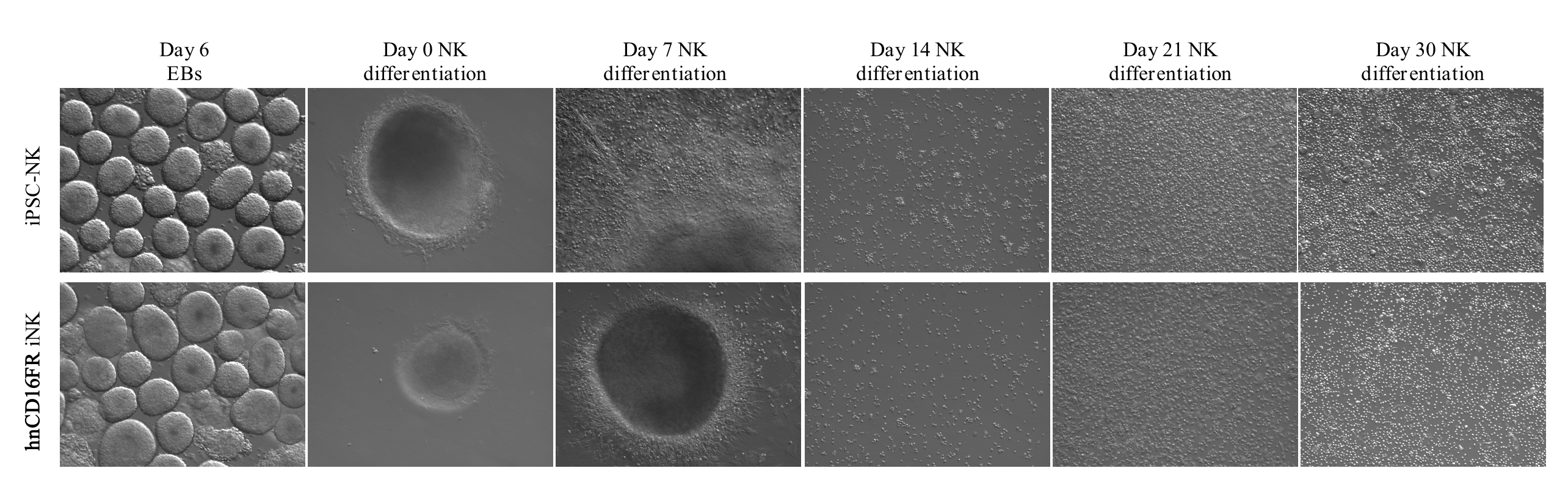

Supplement: Supplementary file 4 — Additional file 4. Fig. S4: Cell images during differentiation of NK cells from human pluripotent stem cells at different stages. [file 13045_2023_1455_MOESM4_ESM.png]

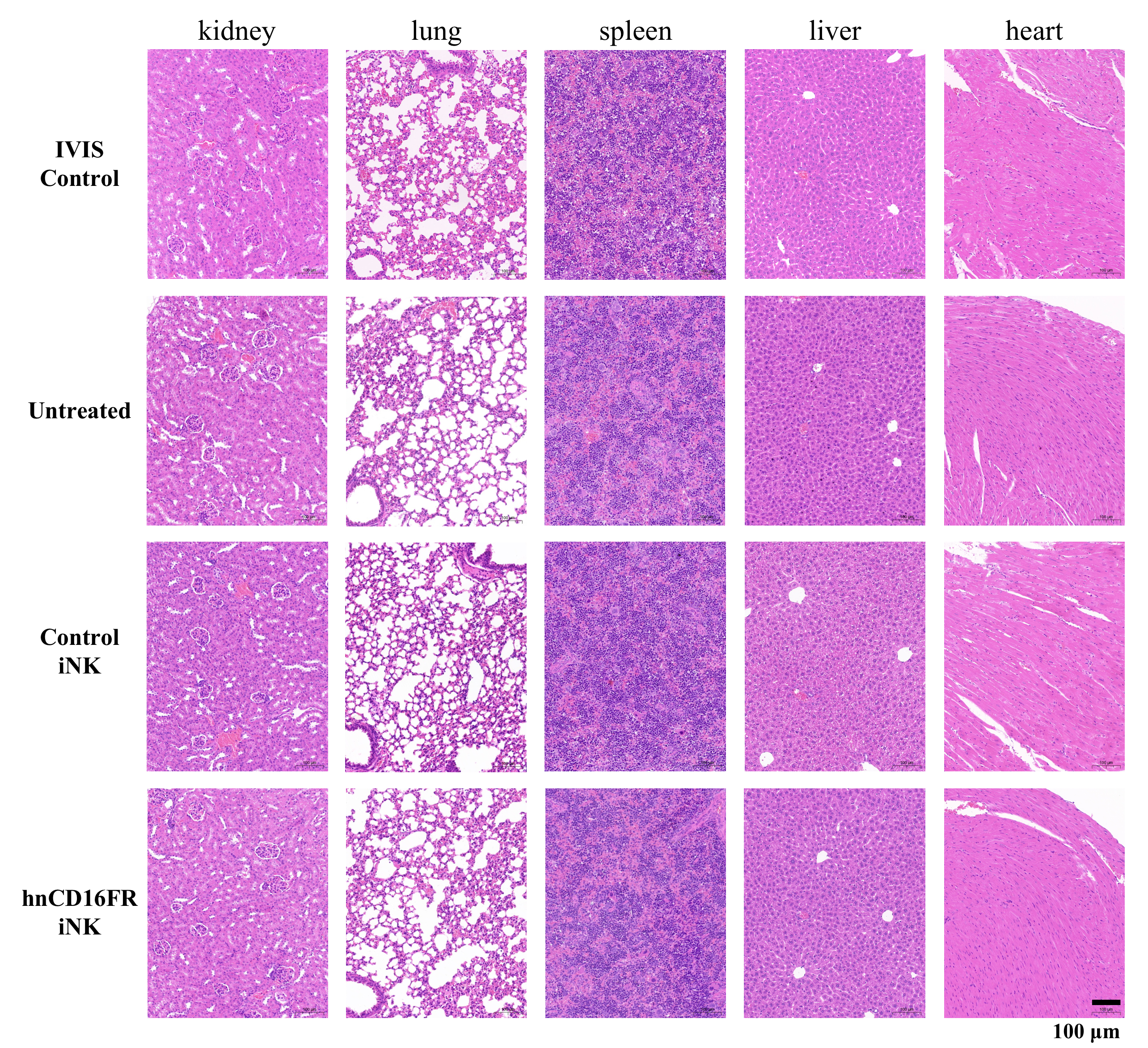

Supplement: Supplementary file 5 — Additional file 5. Fig. S5: Representative images of H&E-stained sections of five major organs 7 days after iNK cell infusion. Representative tissue images of kidney, lung spleen, liver, and heart histopathology from mice receiving only tumor cells, control iNK cells, or hnCD16FR iNK cells. Scare bar is shown as 100 μm. [file 13045_2023_1455_MOESM5_ESM.png]
